# CLEC4E deletion in cardiac resident cells reduces infarct size and improves cardiac remodeling following myocardial ischemia-reperfusion injury

**DOI:** 10.3389/ebm.2026.11196

**Published:** 2026-10-26

**Authors:** Michiel Algoet, Ming Wu, Hilde Gillijns, Matic Pusovnik, Ellen Caluwe, Kasia Blazejczyk, Willy Gsell, Lotte Geerkens, Senne De Groote, Tatiana Kuznetsova, Uwe Himmelreich, Wouter Oosterlinck, Stefan Janssens

**Affiliations:** 1 Department of Cardiovascular Sciences, KU Leuven, Leuven, Belgium; 2 Biomedical MRI, Department of Imaging and Pathology, KU Leuven, Leuven, Belgium; 3 Research Unit Hypertension and Cardiovascular Epidemiology, Department of Cardiovascular Sciences, KU Leuven, Leuven, Belgium

**Keywords:** bone marrow chimeric mice, cardiac resident immune cells, CLEC4E, left ventricular remodeling, myocardial ischemia-reperfusion injury

## Abstract

The c-type lectin receptor CLEC4E plays a critical role in innate immune responses to ischemic tissue damage, and is expressed on infiltrating immune cells and tissue resident cells. We investigated whether inhibition of CLEC4E signaling in resident cells or infiltrating immune cells, mediates cardioprotection in myocardial ischemia-reperfusion (I/R) injury, and explored associated molecular pathways. Methods: We generated chimeric mice via sublethal total body irradiation followed by adoptive transfer of WT C57Bl6/J bone marrow cells (BMCs) in CLEC4E^−/−^ mice and CLEC4E^−/−^ BMCs in WT mice (n = 86). Four weeks after BMC transfer, we induced I/R injury by 30 min LAD occlusion. Myocardial necrosis was measured using troponin I (TnI) at 90 min; infarct size and LV remodeling were assessed by gadolinium-enhanced MRI at 24 h and 28 d. Hearts and bones were harvested at 28 d for immunohistochemistry, Olink-based myocardial proteomic analysis, and genotyping. Results: BMC analysis confirmed successful CLEC4E chimerism after transfer of CLEC4E^−/−^ vs. WT progenitors. TnI levels and infarct size at 24 h were similar in both groups, but at 28 d, CLEC4E^−/−^ mice with WT BMCs (n = 26) showed better LV function (LVEF 62% vs. 47%, p < 0.05) and smaller infarcts (6.9% vs. 19.8% LVmass, p < 0.01) than WT with CLEC4E^−/−^ BMCs (n = 22). Proteomic analysis revealed enhanced pro-inflammatory and pro-fibrotic protein expression in WT with CLEC4E^−/−^ BMCs. Conclusion: Deletion of CLEC4E in non–bone marrow–derived cardiac resident cells confers cardioprotection post-I/R. Targeting CLEC4E signaling and its downstream effectors in cardiac tissue may offer a novel therapeutic strategy for I/R injury.

## Impact statement

Our study addresses a significant gap in understanding the innate immune mechanisms that contribute to myocardial injury and repair, with important implications for novel cardioprotective strategies. CLEC4E, a pattern recognition receptor previously implicated in sterile inflammation, has been shown to contribute to ischemia-reperfusion (I/R) injury in brain and myocardial tissue. However, it remains unclear whether CLEC4E expression in infiltrating immune cells or in cardiac resident cells mediates the response to myocardial injury and which molecular pathways are involved. In this study, we have used a robust bone marrow transplantation (BMT) strategy to generate chimeric mice with selective deletion of CLEC4E signaling in hematopoietic cell lineages. By comparing selective deletion of CLEC4E signaling in either cardiac resident or infiltrating immune cell compartments, we demonstrate that inhibition of CLEC4E expression in cardiac resident cells mediates cardioprotection as evidenced by an improved left ventricular function, reduced infarct size, and attenuated adverse remodeling following I/R injury. The structural and functional benefits are supported by proteomic profiling, which identified suppressed pro-inflammatory and pro-fibrotic remodeling pathways in CLEC4E-deficient cardiac resident cells. We believe that our study significantly advances the understanding of myocardial innate immune signaling during I/R injury by: - providing direct in vivo evidence that CLEC4E in cardiac resident cells, rather than in hematopoietic immune cells, drives maladaptive remodeling following I/R, - establishing a mechanistic link between CLEC4E signaling and downstream pathways involved in scar formation, innate immunity, and extracellular matrix remodeling, - proposing cardiac-resident CLEC4E as a viable therapeutic target for future drug development in ischemic heart disease.

## Introduction

Cardiac repair following myocardial infarction (MI) requires a highly coordinated and complex process first characterized by the infiltration of immune cells [1, 2], and subsequently by a transition into a reparative and proliferative phase [3]. A timely and balanced regulation of the early inflammatory response is critical to promote healing while preventing maladaptive left ventricular (LV) remodeling, poor scar formation, and adverse outcomes [4–6]. Excessive or prolonged inflammation can lead to ventricular dilation, systolic dysfunction, and an increased risk of heart failure while an insufficient inflammatory response impairs clearance of necrotic cells and extracellular matrix components, thereby hindering tissue repair. A better understanding of the cellular and molecular mechanisms that govern this biphasic immune response is required to develop next-generation therapies for cardiac repair following ischemia-reperfusion (I/R) injury [6].

The C-type lectin domain family 4 member E (CLEC4E) receptor plays a crucial role in innate immunity by initiating signaling pathways that regulate cytokine production, pathogen clearance, and adaptive immune responses. The CLEC4E receptor was initially found to be expressed on leukocytes and macrophages in response to bacterial pathogens and fungi [7]. However, it soon became clear that CLEC4E also recognizes necrotic material as a ligand and was reported to be upregulated not only in infiltrating inflammatory cells, but also in neuronal and cardiac tissue cells following reperfusion injury [8] We previously reported reduced acute cardiac injury and improved LV remodeling at 4 weeks post-injury in mice with targeted deletion of CLEC4E gene function (CLEC4E^−/−^) after myocardial I/R compared to wild type mice [9], but whether cardioprotection is mediated by inhibited CLEC4E signaling in (cardiac) resident cells or infiltrating immune cells and which downstream effectors are involved, remains unknown.

The aim of the present study is thus twofold: first, to generate bone marrow chimera to determine whether CLEC4E gene function in (cardiac) resident cells versus in infiltrating immune cells mediates cardioprotection; second, to perform myocardial tissue analysis and explore underlying downstream pathways, which may signal novel therapeutic targets for myocardial repair.

## Materials and methods

### Ethics

All animal procedures were performed according to the Belgian law on care and use of experimental animals and were approved by the Ethics Committee for Animal Experimentation at KU Leuven (P146/2021).

### Bone marrow transplantation

Eight- to nine-week-old male C57Bl6/J wild-type (WT) and C57Bl6/J CLEC4E^−/−^ mice received a bone marrow cell transplantation from female donor mice (Supplementary Figure 1). To control for the effect of the BM transplantation procedure, an additional group of WT mice was included that underwent the same myeloablation but was reconstituted with WT bone marrow cells and this group is referred to as control (CON). After euthanasia, femoral bones of donor mice were collected for bone marrow cell isolation and 5 × 10^6^ bone marrow cells were suspended in 200 µL serum-free RPMI medium and injected via tail vein in anesthetized, myeloablated mice positioned on a heating plate, as reported by Park et al. [10].

After 3 weeks, mice were re-anesthetized using isoflurane 2.5%, a tail vein blood sample was taken as well as a tissue sample (ear) to determine the genotype and chimerism of the animals by quantitative real-time polymerase chain reaction (qRT-PCR) using TaqMan Gene Expression Assays (Mm01183703_m1, Thermo Fisher Scientific, Belgium) and primers specifically designed to evaluate loss of CLEC4E gene function and sex mismatch. PCR primer sequences and expected amplicon sizes used for CLEC4E (KO) and SRY genotyping are listed in Supplementary Table S1.

### Animal models

Three animal models are studied based on genotype after bone marrow transplantation (BMT).

1. CLEC4E^−/−^ mice transplanted with WT BM: CLEC4E^−/−^ (WT BM)
2. WT mice transplanted with CLEC4E^−/−^ BM: WT (CLEC4E^−/−^ BM)
3. WT mice transplanted with WT BM (control): WT (WT BM)

### Mouse model of I/R injury

Four weeks post radiation and confirmation of successful chimerism, mice underwent I/R injury by 30 min LAD ligation followed by reperfusion, as previously reported [9]. In short, mice were anesthetized with an intraperitoneal (IP) injection of ketamine/xylazine (100 mg/kg and 10 mg/kg, respectively), intubated, and mechanically ventilated with a tidal volume of 7 μL/g BW at 140 strokes/min) (Minivent, Hugo Sachs Elektronik, March-Hugstetten, Germany). After transient LAD ligation and reperfusion, animals were allowed to recover under adequate monitoring and pain relief (Buprenorphine 100 mg/kg). All mice were weighed (Gram) before bone marrow transfer, before ischemia-reperfusion, and before euthanasia. Body Surface Area (BSA, cm^2^) was calculated using the following formula: BSA (cm^2^) = 9.82 × (body mass in grams)^(2/3) [10].

### Cardiac MRI

#### Late gadolinium enhancement (LGE) cardiac magnetic resonance (CMR) image acquisition

Cardiac MRI was performed at 24h (infarct size, functional parameters) and at 4 w post reperfusion (functional parameters) on a Bruker BioSpec 70/30 7T MRI system equipped with actively shielded gradients (200 mT/m) (Bruker BioSpin) by investigators blinded to the genotype.

An 86 mm quadrature transmit coil combined with a rat brain surface receive-only coil (T20010V3, Bruker PCI) placed over the chest of the animals was used with the animals positioned supine. Inhalation anesthesia was maintained at 1.5% isoflurane in 1.5 L/min O_2_ throughout all MRI experiments. Body temperature, respiration and heart rate were maintained at 36–37 °C, 60–90 min^−1^ and 450–600 min^−1^, respectively. Core temperature was maintained using warm water in a closed circuit within the animal bed. ECG signal was derived from three needle electrodes placed in the fore and hind paws. Respiration and body temperature were monitored with a balloon pressure-transducer positioned underneath the animal’s abdomen and a rectal temperature probe, respectively. All cardio-respiratory-temperature monitoring was performed using an MR-compatible system (SA Instruments, NY, USA).

LGE MRI (24 h post-surgery): infarct size (IS) was measured via LGE MRI, 20 min after IP administration of gadolinium contrast (Gd-DOTA, 0.5 mmol/kg) (Dotarem, Guerbet, France, batch: 17GS767B). LGE imaging was performed using a 2D retrospectively gated T_1_-weighted FLASH (IntraGate, Bruker PCI) in axial orientation. Parameters included: TE/TR = 2.3/11.1 ms, flip angle = 45°, matrix = 120 × 110, FOV = 28 × 25 mm^2^, 7–8 contiguous slices (1 mm thickness), and oversampling factor of 200. Images were retrospectively reconstructed into 20 cardiac frames.

Cine imaging (28 days post-surgery): A sequence with parameters matched to LGE-MRI was used, without additional contrast. Imaging data were retrospectively reconstructed into 20 cardiac frames for myocardial function analysis.

#### Image analysis

Functional MRI data was analyzed by a single experienced and blinded investigator (M.A.) in a random order. LGE MRI data was analyzed by two experienced and blinded investigators (M.P. and L.G.) in a random order. Bias field correction was performed using a custom MATLAB (The MathWorks Inc., Massachusetts, United States) script based on code reported by Chunming et al [11, 12]. Next, endocardial and epicardial LV borders in end-diastole were delineated using Segment v4.0 R12067 (Medviso, segment.heiberg.se) [13]. Infarct size (IS) and region of interest (ROI) were quantified in 3 apical slices (excluding the apex) using a threshold-based approach of raw myocardial signal intensity (SI). Manual ROIs were drawn in the septal area to represent the viable myocardium. The software then delineated IS from the remaining part of the myocardium where signal intensities exceeded the threshold by more than 2 standard deviations (SD) from viable ROI. To assess global LV-function and remodeling, ejection fraction (EF) was calculated based on end-systolic volumes (ESV) and end-diastolic volumes (EDV) both during the acute (24 h, LGE-MRI) and chronic (4 w) stage of the remodeling using Segment v4.0 R12067.

$$Infarct\;Size=\sum^{slices}\frac{N\;pixel\;\left(SI>2SD\right)}{N\;pixel\;myocardium}\;*\;100\%$$

### Immunohistochemistry of murine heart tissue

Mice were euthanized via cervical translocation at indicated time points. Left ventricular tissue and femoral bones were collected for histological analysis. Six μm-thick LV transversal sections were prepared from paraffin-embedded tissue after fixation in Zinc Formalin Fixative (Sigma-Aldrich, St. Louis, USA), deparaffinized, and incubated in a neutral antigen retrieval solution (Agilent Technologies, S1699) for 20 min at 95 °C. Endogenous peroxidase was quenched by incubation in 3% H_2_O_2_ in methanol. To determine the cellular origin of CLEC4E expression, sections were co-incubated with rat anti-CLEC4E antiserum (MBL, D292-3M2, dilution 1/50) and mouse anti-alpha-smooth muscle actin-FITC (Sigma-Aldrich, F3777 dilution 1/100, to stain endothelial cells) or rabbit anti-myeloperoxidase (Dako, A039829-2, dilution 1/100, to stain neutrophils). After 24h, sections were washed and incubated with biotinylated donkey anti-rat (Jackson ImmunoResearch, #712-065-153, dilution 1/250) (CLEC4E) and donkey anti-rabbit Alexa488 (Thermofisher, #A-21206, dilution 1/250) (MPO). The biotinylated donkey anti-rat staining was amplified using tyramide signal amplification Cyanine 3 (Akoya Biosciences NEL704A001KT). Additionally, TO-PRO-3 (Life Technologies, # 3605, diluted 1/400) staining was performed to visualize nuclei. To further evaluate CLEC4E expression in cardiac resident cells, CLEC4E-stained sections were incubated with Alexa488-labeled wheat germ agglutinin (Thermo Fisher, W11261, dilution 1/100 to stain endothelial cell membranes) and Alex488-labeled anti-MLC2 antiserum (Abcam, AB48003, dilution 1/100 to stain cardiomyocytes). Images were obtained using a laser scanning confocal microscope (Carl Zeiss, Jena, Germany, LSM 700) and ZEN software. Fibrosis was semi-quantitatively measured on mosaic scans from 9 different Sirius Red-stained sections spanning the area from the LV base to the apex and using AxioVert 200 microscope (Carl Zeiss, Jena, Germany). The presence of collagen was determined using color thresholding (ImageJ software, NIH) and expressed as percentage of Sirius Red-stained area over total tissue area.

### High-sensitivity troponin I ELISA

Hs-TnI levels were determined on plasma samples collected at 90 min reperfusion and at 4 w using ELISA (CTNI-1-HSP, Life Diagnostics, Stoke-on-Trent, United Kingdom) according to the manufacturer’s instructions.

### Bone marrow isolation

Bone marrow cells were collected from femoral bones using the protocol reported by Park et al [14]. In short, bone marrow was collected using PBS, filtered and centrifuged at 310 *g* for 10 min at 4 °C. The cell pellet was resuspended in serum-free RPMI medium and stored at −80 °C until further analysis. Immunohistochemistry for CLEC4E expression was performed on bone marrow smears using specific antisera, as previously described for myocardial tissue sections.

### Proteome analysis

Two proteomic analyses were performed on myocardial tissue samples (whole left ventricle) collected from chimeric mice at euthanasia (28d after I/R injury). Proteins from murine hearts were extracted using RIPA buffer (50 mM Tris-HCl pH 7.4, 150 mM NaCl, 1 mM EDTA, 1% Triton X-100, 0.1% deoxycholic acid) supplemented with Roche Complete Mini protease inhibitor cocktail (Roche #11836153001), 1 mM NaF, and 1 mM Na_3_VO_4_. Protein concentrations were determined using the Bicinchoninic Acid assay, after which all samples were diluted to a final concentration of 0.5 mg/mL and aliquoted to 1 µL per well. The first analysis utilized the Olink Target 48 Mouse Cytokine Panel, which enables absolute quantification of 43 immune-related proteins per well. This panel was applied to myocardial samples collected 4 weeks after I/R injury from 5 chimeric WT mice (CLEC4E^−/−^ BM) and 6 chimeric CLEC4E^−/−^ mice (WT BM). The second analysis used the broader Olink Target 96 Mouse Exploratory Panel, designed for targeted protein biomarker discovery related to cellular stress responses, apoptotic signaling, extracellular matrix organization, cell differentiation and immune signaling. This panel provides relative protein levels of 92 proteins in cardiac tissue extracts, reported as Normalized Protein Expression (NPX) values, which are Log2-transformed from Ct values. This analysis was performed on 4-week post- I/R myocardial samples from 19 chimeric WT mice (CLEC4E^−/−^ BM) and from 14 chimeric CLEC4E^−/−^ mice (WT BM). To ensure unbiased data acquisition, sample randomization was performed using the Olink Analyze R package for both datasets.

### Statistical analysis

Data are presented as mean ± standard deviation. Two groups were compared using Student’s t-test with Welch’s correction where appropriate. Where necessary, Sidak-Bonferroni method was used to correct for multiple comparisons. Three groups were compared using one-way analysis of variance (ANOVA) with Bonferroni’s post-hoc test. Log-Rank test was used to compare survival curves of two groups. Statistical analysis was performed with GraphPad Prism (La Jolla, CA, USA, Version 6.01).

## Results

### Bone marrow chimerism

#### Quantitative real time polymerase chain reaction (qRT-PCR)

Successful BMT was confirmed using qRT-PCR on blood and tissue samples and using immunohistochemistry on bone marrow smears. First, an intermediate qRT-PCR test on tail vein blood samples and ear/tail tissue obtained 3 weeks post BMT shows that male (SRY^+/+^) WT (CLEC4E^+/+^) mice that received adoptive transfer with female (SRY ^−/−^) CLEC4E^−/−^ bone marrow display the CLEC4E^−/−^ genotype in blood samples but not in tissue samples (Figure 1, panel A). Conversely, the absence of the SRY specific transcripts in circulating (blood) cells but not in tissue cells in original male (SRY^+/+^) WT (CLEC4E^+/+^) mice that received female (SRY^−/−^) CLEC4E^−/−^ bone marrow cells confirms successful BMT (Figure 1, panel B).

**FIGURE 1:**
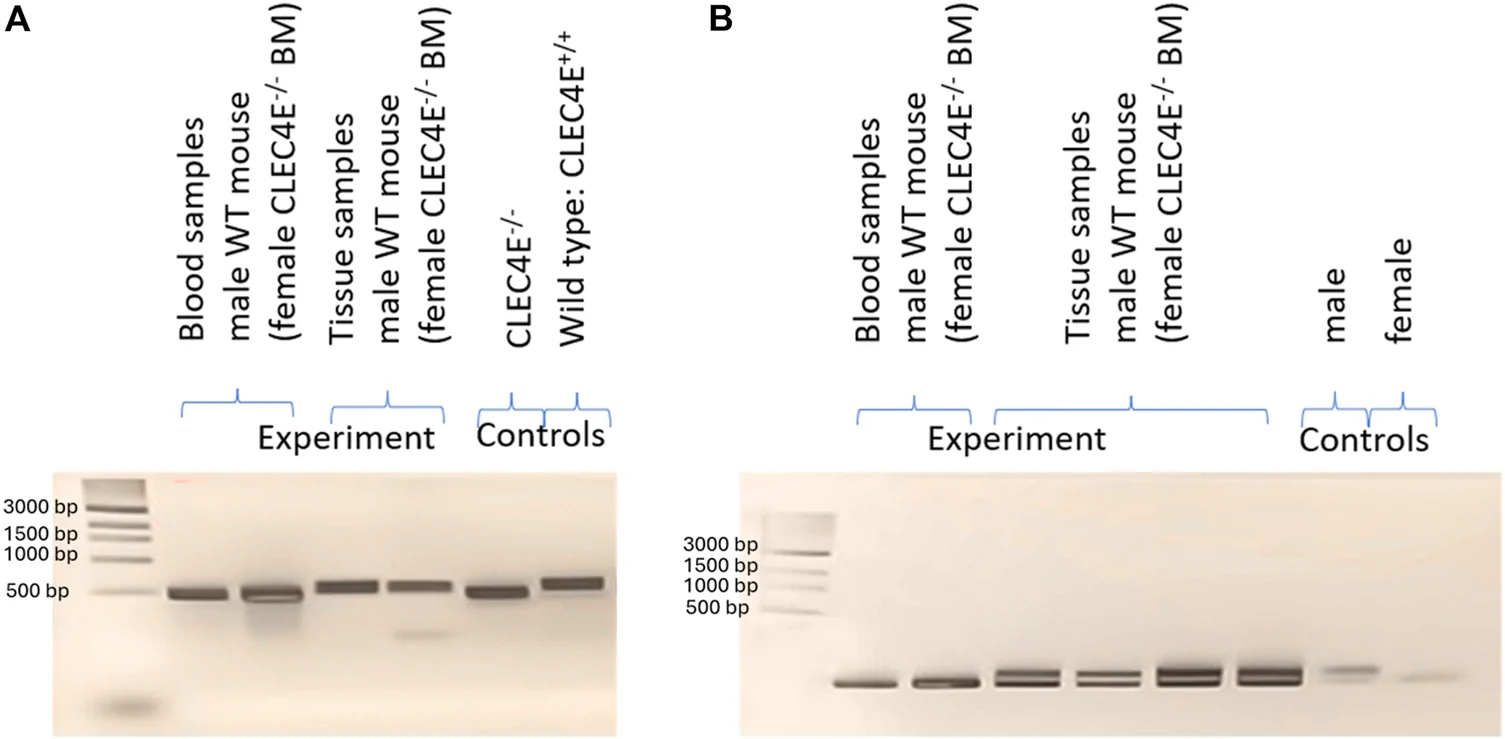
Confirmation of successful adoptive bone marrow transfer using PCR-based genotyping. **(A)** PCR amplification of the CLEC4E locus demonstrates the CLEC4E^−/−^ genotype in circulating blood cells but not in tissue cells of male WT recipient mice, confirming donor-derived hematopoietic reconstitution (1kb DNA ladder. Expected amplicon sizes: 488 bp (mutant) and 593 bp (wild type). **(B)** PCR amplification of the SRY locus shows absence of the Y-chromosome–specific band in circulating blood cells derived from female donor mice, while remaining detectable in tissue cells of male WT recipients (1 kb DNA ladder. Expected amplicon size: ∼120 bp). PCR primer sequences CLEC4E (KO) and SRY genotyping are listed in Supplementary Table 1.

#### Bone marrow smear

Second, immunohistochemical analysis of CLEC4E expressing cells in bone marrow smears confirmed successful bone marrow transfer (Figure 2). At baseline, the average percentage of CLEC4E expressing bone marrow cells from wild type (CLEC4E^+/+^) and CLEC4E^−/−^ mice was 89% (SD ± 5.61%) and 3% (SD ± 1.18%), respectively (panels A, B). WT mice that received adoptive transfer with CLEC4E^−/−^ BM cells show 7% (SD ± 3.56%) CLEC4E immunoreactivity in BM smear compared to >99% (SD ± 0.51%) in bone marrow cells of CLEC4E^−/−^ mice that received WT BM (panels C, D) (p < 0.01, n = 10).

**FIGURE 2:**
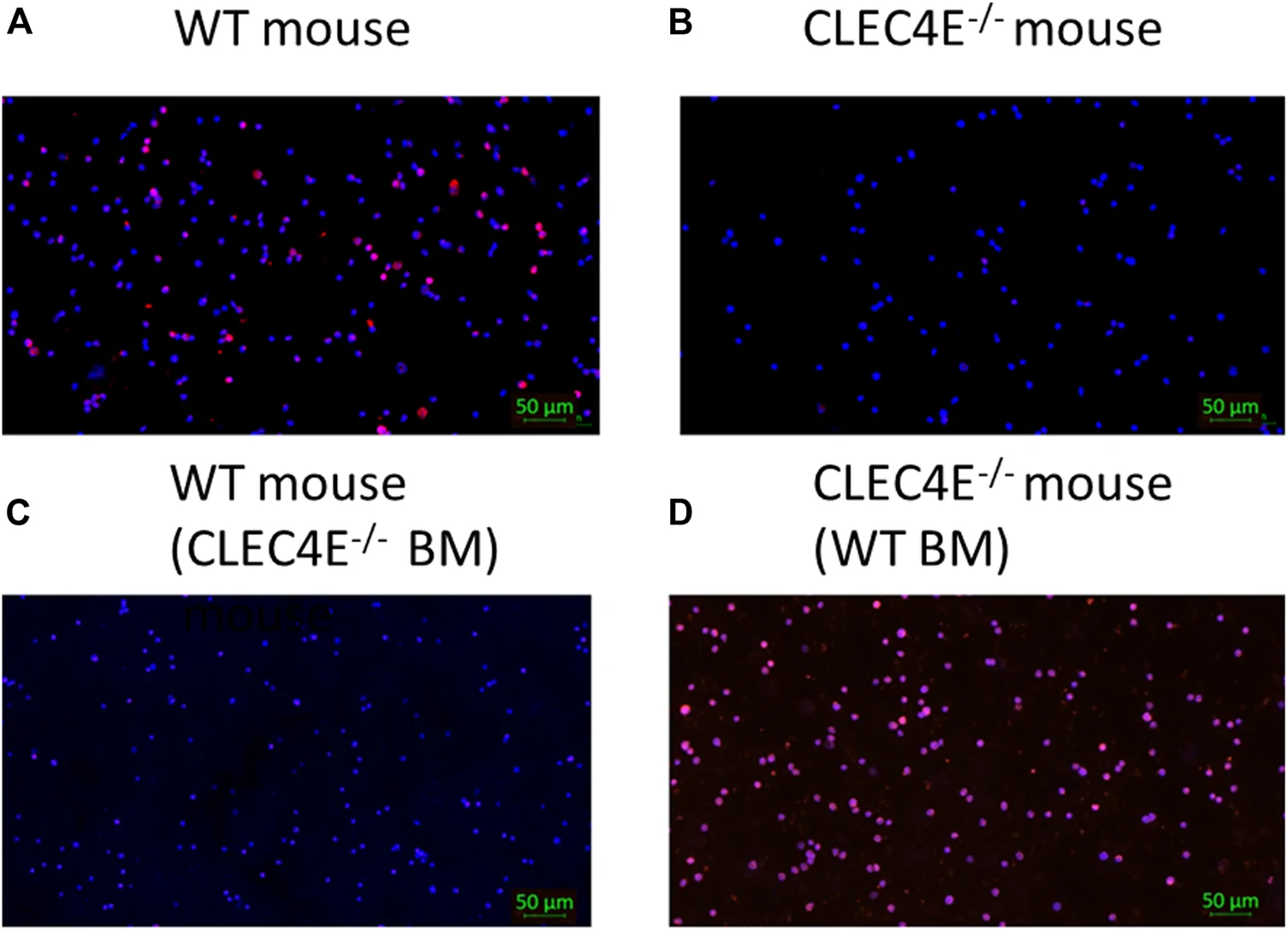
Immunohistochemistry of bone marrow smears. Cell nuclei are stained blue (TOPRO); red (Cy3) immunofluorescence represents CLEC4E expression. Panels **(A,B)** represent bone marrow smears of representative wild type and a CLEC4E^−/−^ mouse, respectively. Panels **(C,D)** represent bone marrow smears of chimeric mice. Scale bar 50 µm.

#### Myocardium

A subgroup of chimeric animals was euthanized at 24h post myocardial I/R to assess the early neutrophil-mediated inflammatory response in the reperfused myocardium using a double staining for MPO and CLEC4E immunoreactivity (Figure 3). In CLEC4E^−/−^ mice with WT bone marrow, no CLEC4E immunoreactivity is detected in the non-infarcted myocardium, whereas the infarcted region shows intense CLEC4E immunoreactivity attributable to infiltrating MPO-expressing inflammatory cells. In contrast, WT mice with CLEC4E^−/−^ BM show uniform distinct CLEC4E immunoreactivity throughout the myocardium but no immunoreactivity in infiltrating neutrophils.

**FIGURE 3:**
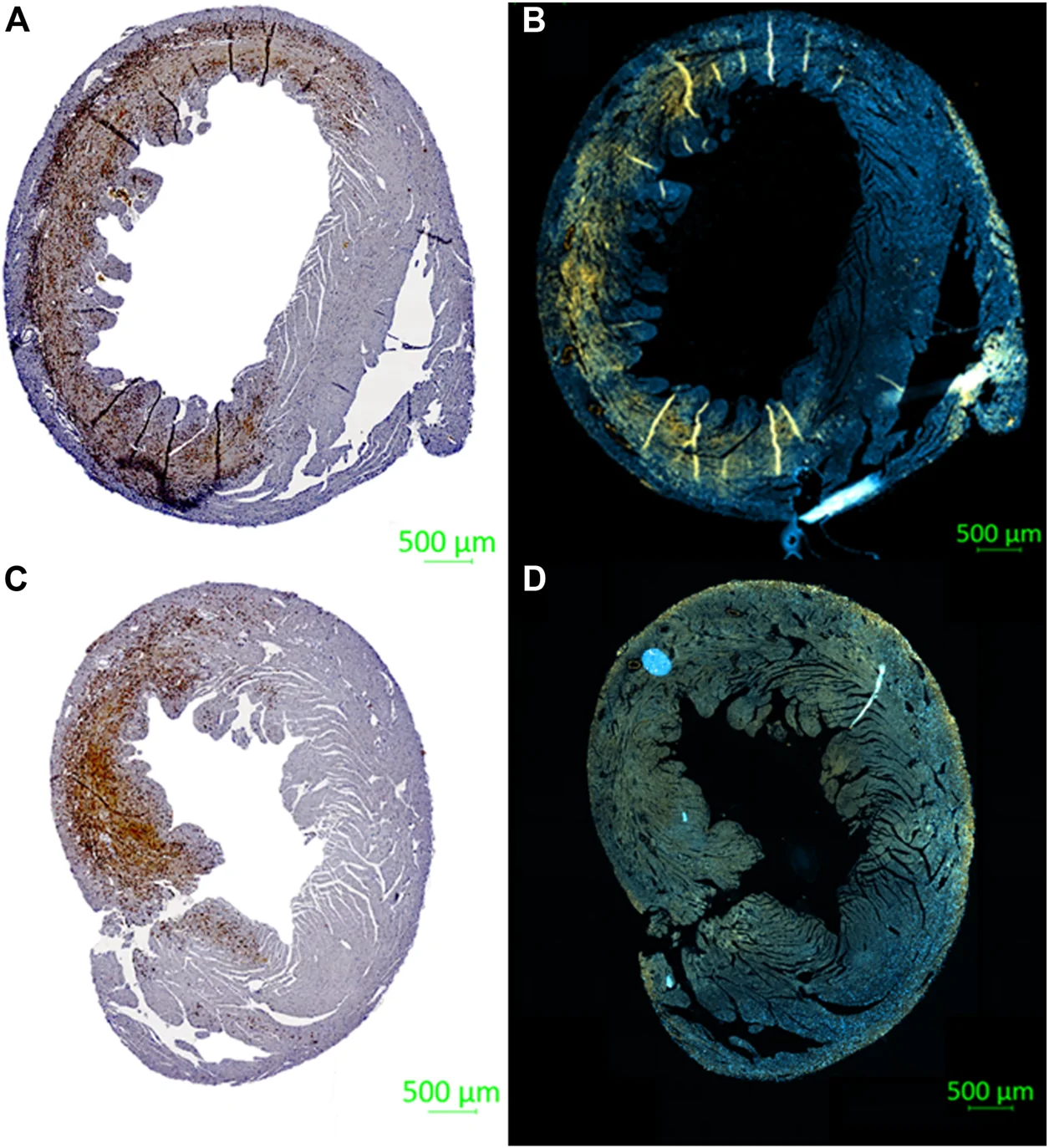
Myeloperoxidase and CLEC4E expression in the infarct zone of a CLEC4E^−/−^(WT BM) mouse **(A,B)** and of a WT (CLEC4E^−/−^ BM) mouse **(C,D)**. CLEC4E (yellow) is expressed in the same area as MPO-expressing WT neutrophils that infiltrate the infarcted region of CLEC4E^−/−^ mice, whereas WT mice that received CLEC4E^−/−^ bone marrow (panels C and D) express CLEC4E throughout the myocardium and not restricted to the infarct region infiltrated by MPO-expressing neutrophils. Left panels **(A,C)**: light microscopic images of MPO staining. Right panels **(B,D)**: immunofluoroscopic images showing CLEC4E (yellow), and TOPRO (blue).

### Localization of CLEC4E expression after myocardial ischemia/reperfusion injury in mice

To identify CLEC4E-expressing cells in the heart, double immunofluorescence was performed at 24 h after I/R using cell-type specific antisera: myosin light chain 2 (MLC2) antiserum for cardiac myocytes, lectin (FITC) for endothelial cells and myeloperoxidase (MPO) for neutrophils. In WT mice (CLEC4E^−/−^ BM) we observed intense CLEC4E immunoreactivity throughout the infarct zone. Co-staining of CLEC4E was observed with MLC2 and lectin (FITC-labeled), but not with MPO. Modest CLEC4E expression was observed throughout the infarct zone of CLEC4E^−/−^ mice and distinct co-immunoreactivity with MPO indicated this was originating from WT inflammatory cells, whereas no co-staining was detected with MLC2 or lectin (Figure 4).

**FIGURE 4:**
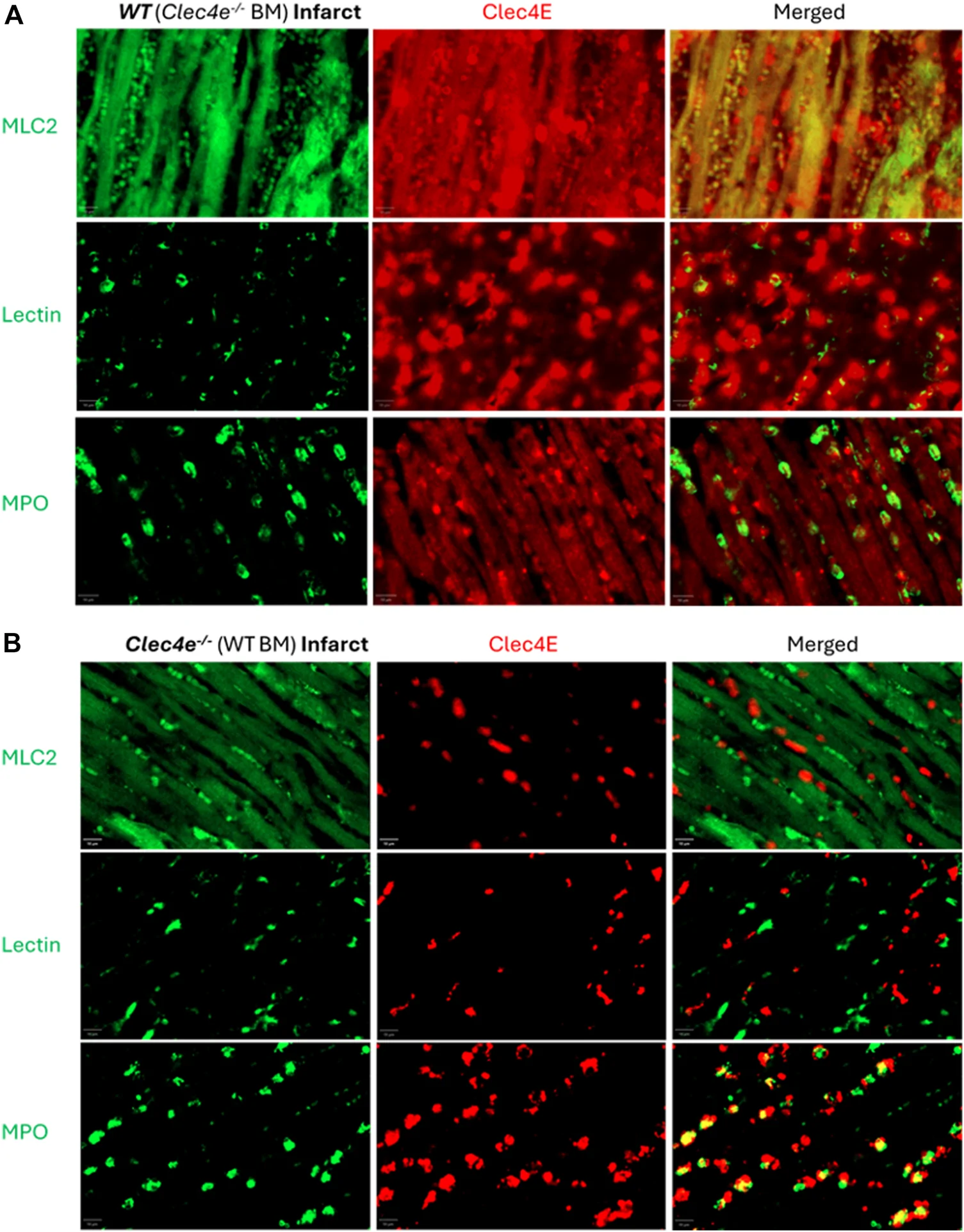
Localization of CLEC4E in heart tissue 24 h after myocardial I/R mice by double-immunostaining of CLEC4E and marker proteins. Panel **(A)**, infarct zone of WT (CLEC4E^−/−^ BM) mouse. Panel **(B)**, infarct zone of CLEC4E^−/−^ (WT BM). Green: MLC2, Lectin or MPO, red: CLEC4E.

### Infarct size in chimeric mice at 24 h and 28 d after I/R injury

Bone marrow chimerism for CLEC4E gene function or the BM transplantation procedure *per se* did not affect the extent of initial ischemic injury as evidenced by similarly increased hs-TnI serum levels at 90 min after reperfusion in chimeric WT mice (CLEC4E^−/−^ BM, n = 22) versus chimeric CLEC4E^−/−^ mice (WT BM, n = 26) versus CON (n = 6) (42 ± 39 vs. 48 ± 62 vs. 44 ± 31 ng/mL, p = NS, respectively). Infarct size measured by LGE MRI 24 h post I/R was also comparable between the 3 groups [31.5 ± 17.0% vs. 25.2 ± 15.6% vs. 31.9 ± 19.4% LVmass in WT (CLEC4E^−/−^ BM) versus CLEC4E^−/−^ mice (WT BM) and CON, p = NS]. In contrast, the subsequent LV remodeling was signifcantly different, infarct size at 4 weeks (semi-quantitatively assessed using planimetry on Sirius Red stains) was significantly smaller in CLEC4E^−/−^ mice (WT BM) than in WT mice (CLEC4E^−/−^ BM) or CON (6.9 ± 12.9% vs. 19.8 ± 13.0% and 22.8 ± 8.9% of LVmass, respectively, p < 0.01) (Figures 5, 6). Taken together, the data clearly demonstrate significantly improved structural remodeling in chimeric mice with loss of CLEC4E gene function in (cardiac) tissue resident cells and rescind any bias or interference potentially introduced by the BM transplantation procedure *per se*.

**FIGURE 5:**
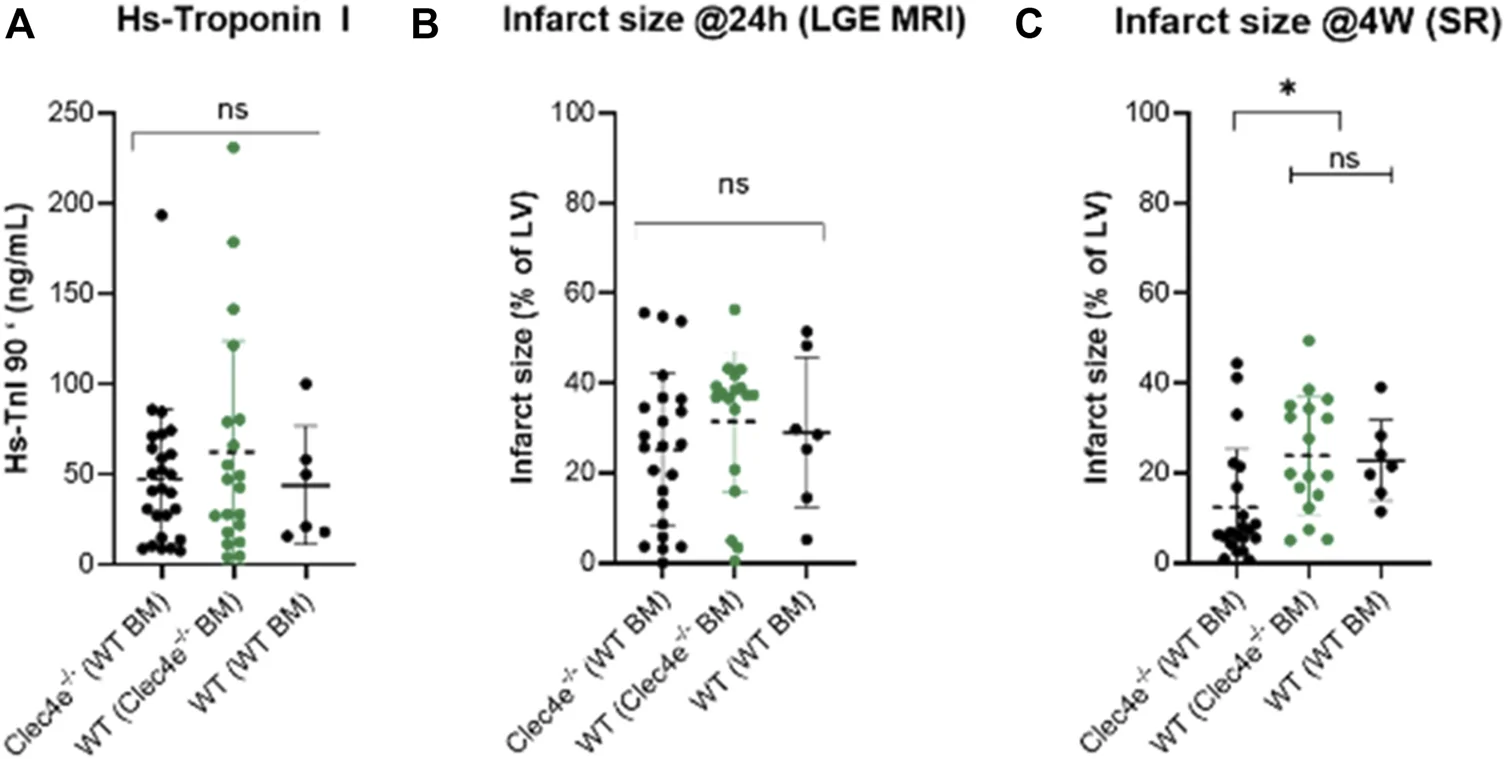
Serum levels of Hs-TnI at 90 min **(A)**, infarct size at 24 h using LGE MRI **(B)** and infarct size at 4 weeks using Sirius Red **(C)** in CLEC4E^−/−^(WT BM), WT (CLEC4E^−/−^ BM) and WT (WT BM) mice after ischemia reperfusion injury, *: p < 0.05.

**FIGURE 6:**
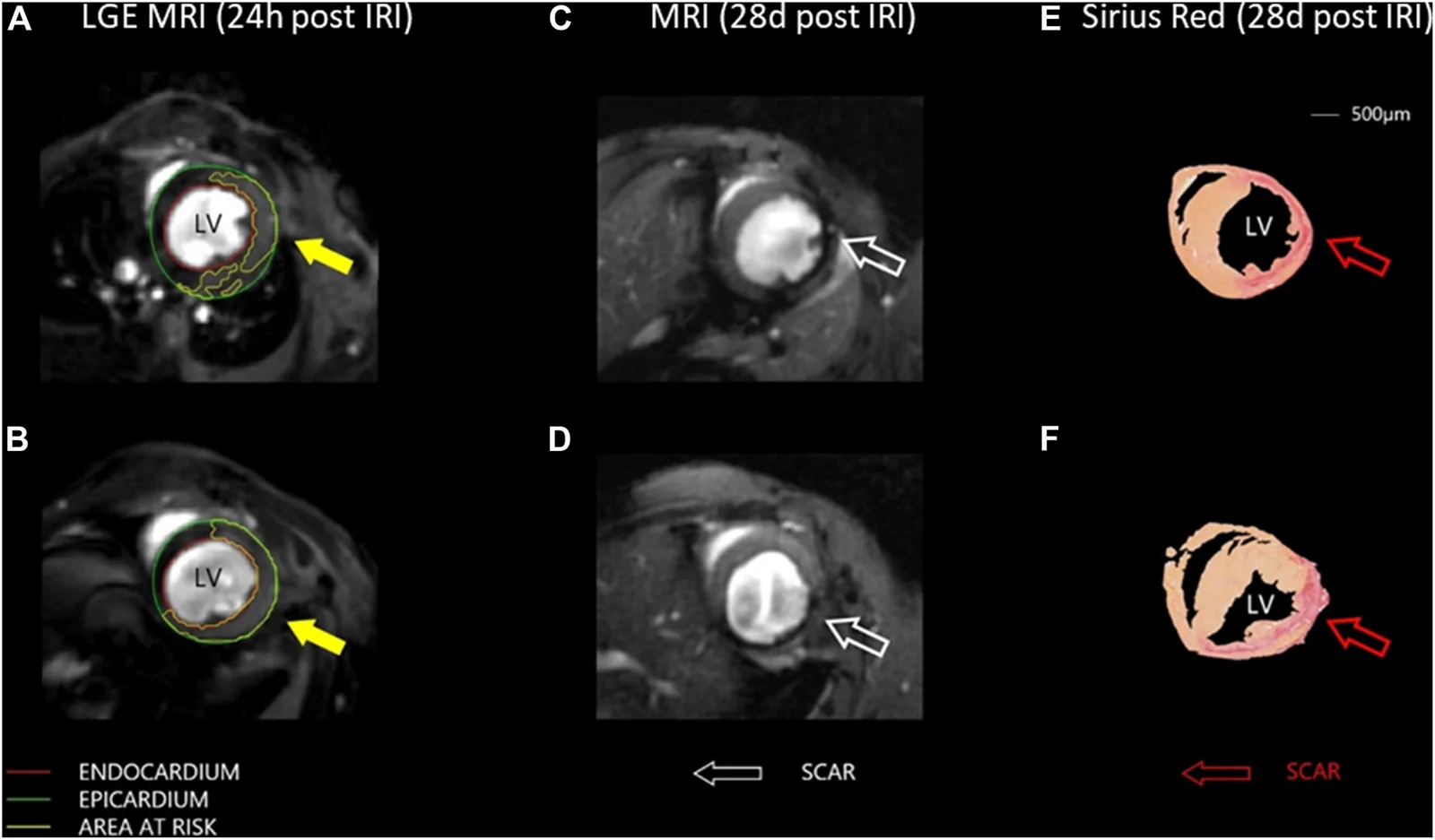
Representative images of myocardial infarct size at 24 h panels **(A,B)** and 28d after panels **(C,D)** I/R as measured by MRI with corresponding scar tissue visualization using Sirius red staining at 28 d panels **(E,F)**. Arrows point toward the infarcted region. Panels **(A,C,F)** illustrate a CLEC4E^−/−^(WT BM) animal; panels **(B,D,E)** illustrate a WT (CLEC4E^−/−^ BM) animal.

### Left ventricular functional remodeling in chimeric mice 28d after I/R injury

After 28 days, LV end-diastolic volumes and end-systolic volumes were significantly smaller in CLEC4E^−/−^ mice (WT BM) than in WT mice (CLEC4E^−/−^BM) (43 ± 13 µL vs. 55 ± 19 μL, p < 0.05//5.1 ± 1.5 mL/m^2^ vs. 6.6 ± 2.2 mL/m^2^, p < 0.05 and 13 ± 8 µL vs. 25 ± 20 µL, p < 0.05//1.5 ± 0.9 mL/m^2^ vs. 3.0 ± 2.4 mL/m^2^, p < 0.05, respectively), resulting in better preserved global LV function (LVEF 62 ± 10% vs. 47 ± 20%, p < 0.05). Functional remodeling at 4 w follow up in the control WT (WT BM) group resulted in a LVEDV of 57 ± 15 µL//6.8 ± 1.8 mL/m^2^, LV ESV of 33 ± 9 µL//3.9 ± 1.1 mL/m^2^ and LV EF of 50 ± 8%. All values of the control group were similar to those observed in WT mice (CLEC4E^−/−^BM) (Figure 7), again refuting any bias introduced by the BM transplantation procedure. During post I/R follow up, LVEF did not change from day 1 to day 28 in CLEC4E^−/−^ mice (WT BM) (Δ LVEF -3 ± 1.6%, p > 0.05) but significantly worsened in WT mice (CLEC4E^−/−^BM) with a mean reduction of −12 ± 5% (p < 0.05) with a similar change in the control WT (WT BM) group (Figure 7).

**FIGURE 7:**
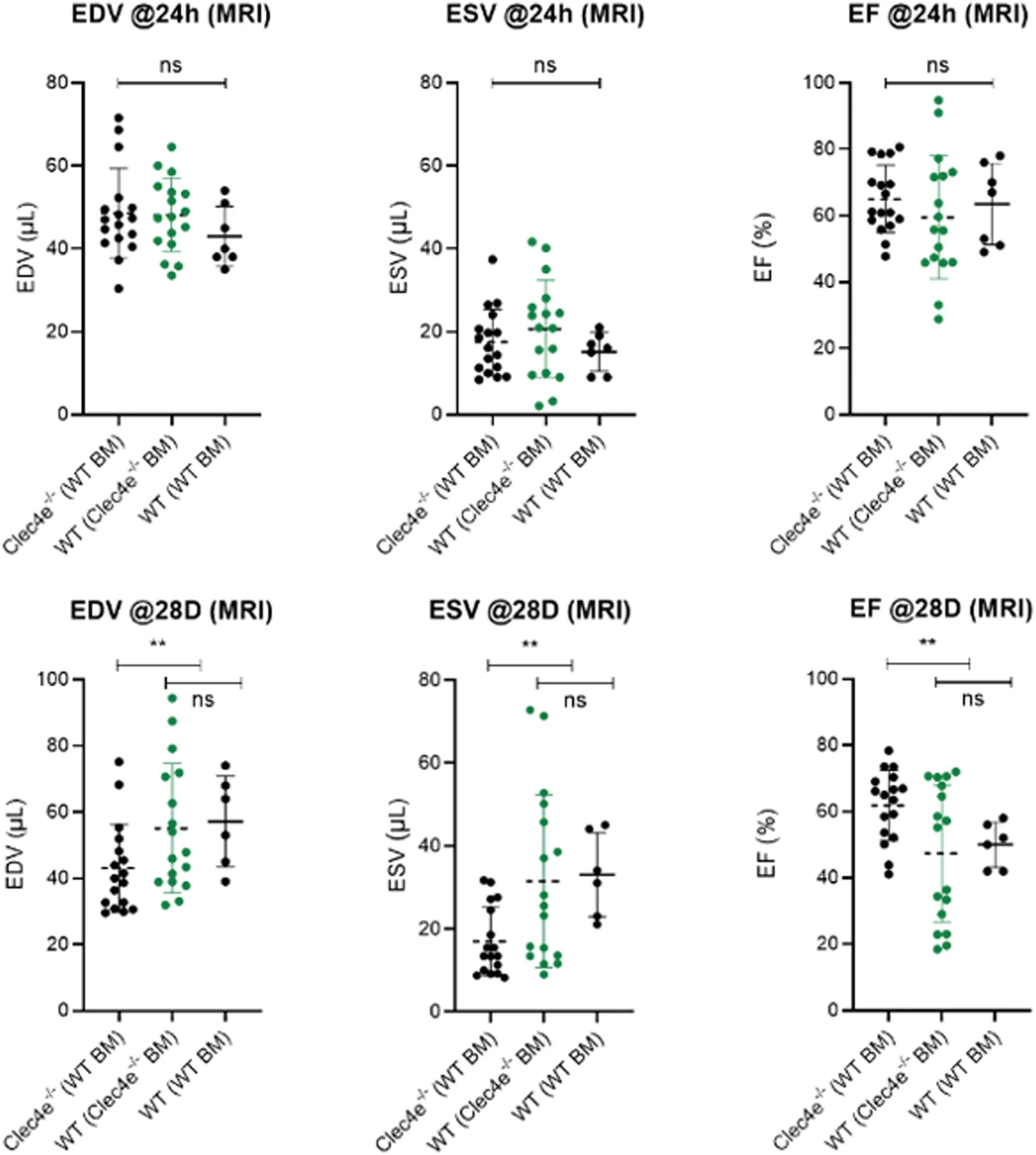
Graphic representation of LV end-diastolic and LV end-systolic volumes (EDV, ESV) and ejection fraction (EF) in CLEC4E^−/−^(WT BM), WT (CLEC4E^−/−^ BM) and WT (WT BM) mice at 24 h (top) vs. 28 days (bottom), **: p < 0.05.

### Proteomics

The first proteome analysis at 4 w post I/R in 5 chimeric WT mice (CLEC4E^−/−^ BM) and 6 chimeric CLEC4E^−/−^ mice (WT BM) included 43 proteins. Three proteins were found to be significantly (p < 0.05) higher expressed in chimeric WT mice (CLEC4E^−/−^ BM), including interleukin 4 (IL-4), Interleukin 1α (IL-1α) and hepatocyte growth factor (HGF) whereas a trend for higher expression was observed for chemokine ligand 12 (CCL12) (p = 0.073) and chemokine ligand 9 (CXCL9) (p = 0.065) (Table 1).

**TABLE 1:** Differentially up- and downregulated proteins in myocardial extracts at 28 days after I/R, determined by direct between-group statistical comparisons using the Olink Target 48 mouse cytokine panel.

Protein symbol	UniProt ID	MEan value CLEC4E^−/−^ (WT BM) (pg/mL)	MEan value WT (CLEC4E ^−/−^ BM) (pg/mL)	p-value	Δ Clec4E^−/−^(WT BM) vs. WT (Clec4E^−/−^ BM)	Biological function
IL-4	P07750	0.18 (±0.09)	0.28 (±0.06)	0.048	+55%	Promoting M2 macro-phage polarization [15]
IL-1a	P01582	0.70 (±0.39)	1.46 (±0.55)	0.025	+108%	Triggers inflammatory response [16]
HGF	Q08048	23.43 (±6.92)	42.7386 (±14.39)	0.016	+82%	Promotes angiogenesis, reducing fibrosis, and improving cardiac function [17]
Ccl-12	Q62401	22.12 (±14.59)	47.46 (±26.39)	0.073	+115%	Recruitment of monocytes and macrophages [5]
CXCL-9	P18340	17.96 (±10.28)	36.09 (±18.07)	0.065	+101%	Proliferation and migration of fibroblasts [14, 18]

Absolute protein concentrations are reported in standard units (pg/mL).

A second proteomics analysis was done on 4 weeks post I/R samples of 21 chimeric WT mice (CLEC4E^−/−^ BM) versus 14 chimeric CLEC4E^−/−^ mice (WT BM). Seven proteins were found to be differentially expressed between both groups: transforming growth factor α (TGF-α) was significantly downregulated in WT (CLEC4E^−/−^ BM), whereas Axin-1 (AXIN1), Cysteine-rich angiogenic inducer 61 (CYR61), Hepatocyte growth factor (HGF), Serine/threonine-protein kinase PAK 4 (PAK4) and Poly [ADP-ribose] polymerase 1 (PARP-1) were all significantly higher expressed in WT (CLEC4E^−/−^ BM). In contrast, Troponin I3 (TNNI3) expression was significantly higher in CLEC4E^−/−^ mice (WT BM). Integrin Subunit Beta 1 Binding Protein 2 (ITGB1BP2) (p = 0.071) and tumor necrosis factor ligand superfamily member 12 (TNFSF12) (p = 0.058) showed a trend for higher relative expression in WT mice (CLEC4E^−/−^ BM) (Table 2).

**TABLE 2:** Relative abundance of differentially up- and downregulated proteins in myocardial extracts at 28 days after I/R, determined by direct between-group statistical comparisons using the Olink Target 96 mouse cytokine panel.

Protein symbol	UniProt ID	MEan value CLEC4E^−/−^ (WT BM) (NPX)	MEan value WT (CLEC4E^−/−^ BM) (NPX)	p-value For Δ NPX	Δ Clec4E^−/−^(WT BM) vs. WT (Clec4E^−/−^ BM)	Biological function
TGFA	P48030	−1.34 (±0.26)	−1.68 (±0.29)	<0.001	+27%	Tissue repair and scar formation [7, 19]
Axin1	O35625	3.03 (±0.50)	3.49 (±0.62)	0.028	−28%	Influences fibrosis, inflammation and myocyte proliferation [20]
Tnni3	P48787	13.15 (±0.34)	12.52 (±0.47)	<0.001	+55%	Influences cardiac remodeling by affecting calcium-mediated processes [6]
CYR61	P18406	1.79 (±0.47)	2.45 (±0.63)	0.003	−37%	Promotes the formation of a collagenous scar in the infarct area [21]
HGF	Q08048	1.68 (±0.51)	2.16 (±0.73)	0.042	−29%	Promotes angiogenesis, reducing fibrosis, and improving cardiac function [17]
PAK4	Q8BTW9	1.06 (±0.41)	1.48 (±0.43)	0.016	−25%	Promotes breaking down the extracellular matrix of the scar. [17]
PARP1	P11103	3.39 (±0.41)	3.84 (±0.65)	0.035	−27%	Adverse left ventricular remodeling [8]
Itgb1bp2	Q9R000	7.74 (±0.31)	7.94 (±0.30)	0.071	−13%	Promotes fibrosis by influencing the extracellular matrix [9]
Tnfsf12	O54907	2.25 (±0.45)	2.60 (±0.54)	0.058	−22%	Adverse left ventricular remodelling [22]

Data are reported as normalized protein expression (NPX), referring to Olink’s relative protein quantification unit on log2 scale, with higher numbers representing higher protein levels in the sample. The difference between NPX values in Clec4E^−/−^ and WT mice is a log2-scaled relative abundance value and these differences on a log 2 scale were back-transformed (2^(Delta NPX)) to obtain the fold change (increase or decrease) in relative protein abundance between both groups.

## Discussion

### Bone marrow chimera strategy to decipher CLEC4E function in I/R injury

In this study, we characterized a robust bone marrow transplantation (BMT) model to investigate the CLEC4E receptor-mediated innate immune response to cardiac ischemic injury and observed that cardioprotection against I/R injury is attributable to loss of CLEC4E expression in non bone marrow derived (cardiac) resident cells, rather than in infiltrating hematopoietic immune cells. We confirmed high-level chimerism using qRT-PCR and immunohistochemical analysis with CLEC4E being expressed in 99% of bone marrow cells in chimeric CLEC4E^−/−^ mice receiving WT bone marrow, compared to only 7% in chimeric WT mice receiving CLEC4E^−/−^ bone marrow. Chimeric CLEC4E^−/−^ mice had a significantly higher LV ejection fraction 28 days after reperfusion injury, an effect that was predominantly mediated by a better preserved systolic function as evidenced by a twofold smaller end-systolic volume compared to chimeric WT mice. Consistent with improved functional remodeling, chimeric CLEC4E^−/−^ mice also showed significantly better structural remodeling evidenced by significantly smaller infarcts than WT with CLEC4E^−/−^ BMCs (6.9% vs. 19.8% LVmass, respectively, p < 0.01). The observed improvement in myocardial repair in chimeric CLEC4E^−/−^ mice are reflected in the targeted proteome findings of decreased expression of proteins involved in pro-inflammatory and pro-fibrotic signaling, including IL1α, IL4, TGFα, Pak4, and Axin 1. Of note, the BMT procedure *per se* did not affect the initial myocardial injury or remodeling process at 28 days as evidenced by similar outcomes in WT mice undergoing WT BMT. Taken together, our data indicate the important role of CLEC4E innate immune receptor signaling in (cardiac) resident cells, and suggest that targeted interventions that block CLEC4E signaling in the myocardium may mitigate infarct extension and adverse left ventricular remodeling following I/R.

### CLEC4E in cardiac resident cells modulates LV remodeling, not initial injury

Myocardial remodeling is a critical process following I/R injury, encompassing changes in size, shape, and function of the heart. While this process aims to repair damage, negative or maladaptive remodeling can lead to heart failure. CLEC4E is implicated in modulating the balance between repair and pathological remodeling by influencing immune cell behavior and extracellular matrix (ECM) turnover. We previously analyzed the temporal changes in the transcriptome of circulating white blood cells in murine and porcine models of myocardial injury and in patients with acute myocardial infarction (AMI) and observed that significant early activation of CLEC4E was associated with impaired healing and LV dysfunction at follow-up [9]. Prior studies in CLEC4E^−/−^ mice in our laboratory have revealed that absence of this receptor in both circulating immune cells and resident tissue cells interferes with myocardial neutrophil infiltration and Cxcr2 receptor sensitivity leading to reduced fibrosis and better-preserved cardiac function after I/R injury [9] Building on these initial findings, our study was designed to understand whether CLEC4E signaling in either circulating immune cells or resident cardiac cells mediates the observed cardioprotection and which downstream signaling pathways are involved.

Both CLEC4E^−/−^ (WT BM) and WT (CLEC4E^−/−^ BM) mice showed comparable plasma hs-TnI levels at 90 min and infarct sizes measured using MRI at 24 h post-reperfusion. These findings suggest that the extent of very initial ischemic injury is independent of CLEC4E gene function. In contrast, infarct size at 4 weeks was significantly smaller in CLEC4E^−/−^ (WT BM) mice with better preserved LV systolic function and attenuated LV dilation. These findings suggest that CLEC4E gene function in non–bone marrow–derived cells, and in particular for this study in cardiac resident cells plays an important role in mitigating adverse structural and functional remodeling, independent of infiltrating immune cell genotype.

To further investigate the spatial distribution and cellular origin of CLEC4E expression in cardiac resident cells following ischemic injury, we performed extensive immunohistochemical studies using confocal analysis and observed that in the early post I/R phase (i.e. 24 h) CLEC4E co-localizes with endogenous cardiomyocytes (MLC2) and endothelial cells (Lectin) in the infarct area of WT chimeric mice in whom hematopoietic cell progenitors and their progeny, including neutrophils, lack CLEC4E gene function. Similar observations were reported by Suzuki et al in the context of ischemic stroke with induction on CLEC4E expression in murine neuronal cells in response to dying brain cells [8].

### Proteomic analysis supports differential remodelling pathways

To explore underlying molecular mechanisms and downstream pathogenic signaling pathways by which CLEC4E modulates myocardial remodeling responses after ischemic injury, we performed differential proteomic analysis of myocardial extracts at 28d after I/R injury in both chimeric groups. Olink-based proteomic profiling further differentiated the molecular landscapes. Using the quantitative Olink Target 48 Mouse Cytokine Panel, we identified three significantly upregulated proteins in WT mice (CLEC4E^−/−^ BM) which had a significantly less favorable post I/R injury course. The marked upregulation of the potent pro-inflammatory IL- 1α cytokine contributes to an exaggerated inflammatory response resulting in adverse cardiac remodeling [16, 23]. The trend for higher expression of CCL-12 and CXCL-9 also supports this pro-inflammatory phenotype. In contrast, IL-4 modulates inflammation by promoting the polarization towards a reparative M2 phenotype and is therefore associated with a counter-regulatory role in adverse cardiac remodeling, as is the case for HGF [21]. Upregulation of IL-4 and HGF may reflect compensatory anti-inflammatory signaling in the setting of unrestrained injury and follow up studies are warranted to determine the balance between pro-and anti-inflammatory cytokine release and its spatiotemporal distribution [16].

The Olink Target 96 Mouse Exploratory Panel identified 7 significantly differentially expressed proteins. TGF-α, a protein involved in tissue repair after myocardial I/R injury [17, 19], was significantly upregulated in chimeric CLEC4E^−/−^ mice and correlates with the reduced infarct size and better functional remodeling in these mice. Axin-1 is a negative regulator of the Wnt/β-catenin signaling pathway, which contributes to adverse cardiac remodeling after myocardial I/R injury [24], but was significantly downregulated in chimeric CLEC4E^−/−^ mice and associated with better MRI-based measurements of LV functional and structural remodeling at 4w follow up. Troponin I3’s role post myocardial I/R injury is not fully understood yet [25]. The role of CYR61 is associated with regulating collagen alignment in scar tissue and overexpression can lead to excessive fibrosis leading to a larger infarct zone, which is consistent with our observation in chimeric WT mice (CLEC4E^−/−^ BM) [26]. Hepatocyte growth factor was found to be upregulated in WT mice (CLEC4E^−/−^ BM), which could signal a counterregulatory phenomenon to more extensive damage [16]. Serine/threonine-protein kinase PAK 4 is involved in this inflammatory process, and its activation can lead to an increased scar tissue formation [16], as observed in WT mice (CLEC4E^−/−^ BM). The PARP1 protein drives cardiomyocyte proliferation and scar formation, and its inhibition has been shown to improve cardiac function and reduce fibrosis [27]. Upregulation of PARP1 in contrast drives adverse cardiac remodeling, as observed in our WT mice (CLEC4E^−/−^ BM) [22]. Finally, the trends for higher protein abundance of ITGB1BP2 (excessive fibrosis) and TNFSF12 (adverse left ventricular remodeling) in chimeric WT mice (CLEC4E^−/−^ BM),is again consistent with our observations of a larger infarct size and worse cardiac remodeling than in chimeric Clec4E^−/−^ mice [28, 29]. Taken together, the collective proteome data are consistent with our experimental findings of improved functional and structural cardiac remodeling in CLEC4E^−/−^ mice (WT BM), which is predominantly accounted for by proteins that regulate scar formation.

### Reinterpreting the role of hematopoietic CLEC4E

Contrary to prior reports suggesting that CLEC4E on infiltrating immune cells has an important injurious role in sterile inflammation post-I/R [20, 30], our data show that its deletion in hematopoietic cells alone (WT mice + CLEC4E^−/−^ BM) is insufficient to confer protection. These mice displayed worse functional outcomes, larger infarcts, and more adverse remodeling. Thus, our findings suggest a model in which CLEC4E in resident cardiac stromal or vascular cells orchestrate the pro-fibrotic and maladaptive remodeling response, potentially by modulating the local cytokine milieu and fibroblast activation. This hypothesis has been explored by Arumugam et al [31] in ischemic central nervous system injury, where they reported that blocking CLEC4E signaling in brain tissue itself rather than in circulating peripheral inflammatory cells reduces stroke size and neurological functional outcome.

### Therapeutic implications

Targeting CLEC4E signaling—specifically in cardiac resident cells—may therefore offer a promising therapeutic approach to mitigate maladaptive remodeling post-MI. Given its limited role in early necrotic injury and its profound impact on long-term scar formation and function, CLEC4E represents a strategic molecular target and hinge point for therapeutic intervention. Selective inhibition using small molecule antagonists or blocking antibodies (e.g., anti-CLEC4E monoclonals) may offer promising prospects to reduce excessive fibrosis and better preserve LV function after I/R injury.

### Limitations

We recognize some limitations in our study. First, while our findings highlight a critical role for CLEC4E in cardiac resident cells during I/R injury, the upstream mechanisms responsible for CLEC4E induction in these cells require follow up studies. Unraveling the signaling pathways that trigger CLEC4E expression in ischemic myocardium is beyond the scope of this study but represents an important focus for future fundamental and translational research. Second, gender mismatch was used during bone-marrow transfer to verify engraftment. While no evidence suggests sex-specific differences in inflammatory cell effects on myocardial IRI, we cannot exclude a potential influence on our results. Third, proteomic profiling was performed at a single time point, limiting insight into temporal protein-expression dynamics. Serial sampling and complementary liquid-biopsy analyses will be needed to capture early molecular events and identify circulating cytokines and biomarkers that signal subsequent LV remodeling.Finally, serum markers of innate immune activation were not assessed in this study.

## Conclusion

This study suggests that CLEC4E expression in cardiac resident cells, rather than in hematopoietic immune cells, contributes significantly to post-ischemic LV structural and functional remodeling. In a bone marrow chimera model of myocardial ischemia-reperfusion injury, mice lacking CLEC4E in cardiac resident cells exhibited smaller infarcts, better preserved cardiac function, and a proteomic profile indicative of reduced fibrotic remodeling. In contrast, CLEC4E deletion restricted to immune cells did not yield similar benefits. These findings indicate that CLEC4E in non–bone marrow–derived cells, including cardiac resident cells may play a thus far unrecognized key role in regulating local inflammatory and structural responses after myocardial injury. Targeted modulation of CLEC4E signaling in these cells could represent a potential strategy to limit maladaptive remodeling and support cardiac repair.

## Data Availability

The original contributions presented in the study are included in the article/Supplementary Material, further inquiries can be directed to the corresponding authors.
