# Supplementary material for: CLEC4E deletion in cardiac resident cells reduces infarct size and improves cardiac remodeling following myocardial ischemia-reperfusion injury
Source: Exp Biol Med (Maywood). 2026 Oct 26;251:11196. doi: 10.3389/ebm.2026.11196 (PMC13745377; doi:10.3389/ebm.2026.11196)
Supplement: Supplementary file 1 [file Supplementaryfile1.docx]

***Sup. Fig. 1*** *Schematic overview bone of marrow transplantation and I/R injury model*

***SRY PCR***

*FW: 5' GAT GAT TTG AGT GGA AAT GTG AGG TA 3'*

*Rev: 5 ' CTT ATG TTT ATA GGC ATG CAC CAT GTA 3'*

***CLEC4E qPCR:***

*FW: 5' TCA GTC AAG AAT TGC TGT CCT TT 3'*

*Rev; 5' AGG TCA AGG TTG TCG TAG AGA A 3'*

***CLEC4E KO PCR***

*FW: 5' ATT GCC ACT GAC CCT CCA CC 3'*

*Rev: 5' CCC CTG TCA CTG TTT CTC TGC A 3'*

***Sup. Table. 1*** *PCR primers for SRY, CLEC4E and CLEC4EKO*
